# Anatomical Evidence of Acupuncture Meridians in the Human Extracellular Matrix: Results from a Macroscopic and Microscopic Interdisciplinary Multicentre Study on Human Corpses

**DOI:** 10.1155/2019/6976892

**Published:** 2019-03-21

**Authors:** Norbert Maurer, Helmut Nissel, Monika Egerbacher, Erich Gornik, Patrick Schuller, Hannes Traxler

**Affiliations:** ^1^Johannes Bischko Institut für Akupunktur/Neurologisches Zentrum am Krankenhaus Rosenhügel Wien, 1130 Wien, Riedelgasse 5, Private Practice for General Medicine, Kupelwiesergasse 16, 1130 Wien, Austria; ^2^Johannes Bischko Institut für Akupunktur/Neurologisches Zentrum am Krankenhaus Rosenhügel Wien, 1130 Wien, Riedelgasse 5, Private Practice for Internal Medicine, Schleifgasse 7, 1210 Wien, Austria; ^3^Institute of Histology and Embryology, Department of Pathobiology, University of Veterinary Medicine, Veterinärplatz 1, 1210 Vienna, Austria; ^4^Institute for Solid State Electronics, Vienna University of Technology, Gußhausstraße 25-25a (Gebäude CH), 1040 Wien, Austria; ^5^Center for Anatomy and Cell Biology, Division of Anatomy, Medical University of Vienna, Währinger Straße 13, 1090 Wien, Austria

## Abstract

For more than 2500 years, acupuncture has been applied to support the healing of different diseases and physiologic malfunctions. Although various theories of the meridian system and mechanisms were formulated to explain the functional basis of acupuncture, the anatomical basis for the concept of meridians has not been resolved. The aim of the present study was to search for replicable anatomical structures that could relate to meridians. To this end, four human specimens and additionally two lower legs were dissected anatomically. Our study found evidence that acupuncture meridians were part of the human extracellular matrix and that fascia was an important part of the anatomic substrate of acupuncture meridians. At the same time, we found vessel-nerve-bundles, which were hypothesized to account for 80% of acupuncture points, only in a few acupuncture points. Therefore, our findings contradict the theory that acupuncture points are only located along the nervous channels.

## 1. Introduction

As part of traditional Chinese medicine (TCM), acupuncture has been an energetic and vibrant treatment with a successful application for more than 2500 years [1, 2]. Acupuncture is an ancient aspect of TCM with demonstrated therapeutic effects [2]. By acting (by needles, laser, moxa, pressure, etc.) on certain areas on the surface of the skin, functional disorders can be corrected, and pain can be reduced. Such areas are defined as acupuncture points.

In TCM, meridians are strings connecting acupuncture points, which are considered as passageways through which energy flows throughout the body [1, 2]. The meridian system is composed of 12 principal meridians, each of which connects to an organ system and extends to an extremity and eight collaterals [1–4]. Acupuncture treatments should improve the flow of energy through the meridian network [5–10].

The morphological basis for the concept of meridians in TCM has not been resolved. Recent articles support a relationship between acupuncture points/meridians and fascia [1]. Specifically, anatomical observations of body scan data demonstrated that the fascia network resembles the theoretical meridian system in salient ways, and physiological, histological, and clinical observations support this hypothesis [11, 12].

The aim of the present study was to clarify whether there was macro- and microanatomical substrate of acupuncture meridians.

## 2. Materials and Methods

Four specimens and additionally two lower legs were dissected at the Medical University of Vienna/Division of Anatomy and Cell Biology.

Our aim was to depict vascular nerve bundles of individual acupuncture points in the course of the associated meridian. A vascular nerve bundle (VNB) was defined as a combination of nerves, arteries, veins, and lymphatics in the body that travelled together.

After the initial incision, the skin and subcutis were removed with the help of a scalpel until the superficial fascia was found. Perforating vascular nerve bundles were carefully separated from the subcutaneous adipose tissue. The bursa, such as Bursa patellaris and olecrani, was extirpated. Due to the lack of a superficial fascia in the facial area, the parotid fascia and the facial muscles were defined as the superficial layer.

Since, in most cases, no bundles of vascular nerve bundles were found at the supposed acupuncture points, we determined the acupuncture point* lege artis* on the section preparation and dissected until the panniculus fascia (fascia superficialis corporis). The sites were documented photographically.

The collected tissue samples were fixed in formalin and embedded in paraffin for histological section production. Sections were stained with haematoxylin & eosin (HE) and stained for neuronal expression by immunohistochemical detection of S-100 (DAKO, Glostrup, DK, dilution 1:1200, detection substrate DAB).

## 3. Results

**Figure 1:**
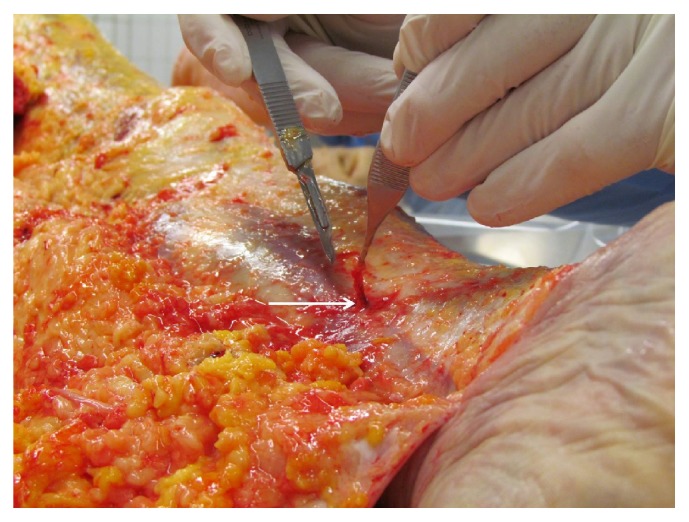
VNB distal to the medial side of the left lower leg.

**Figure 2:**
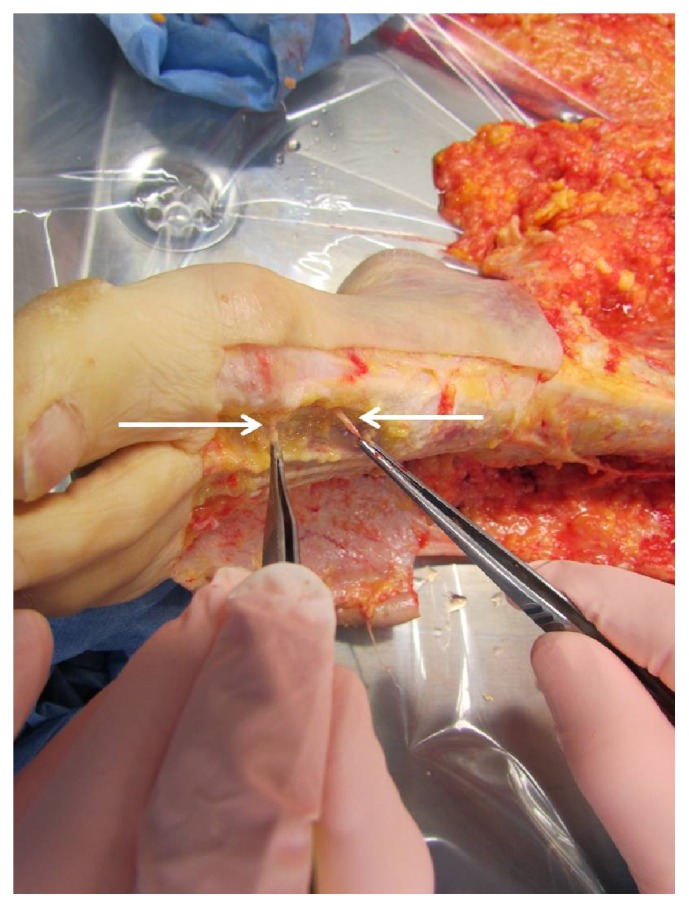
VNB at the acupuncture point and proximal of it without acupuncture point on the left dorsum.

**Figure 3:**
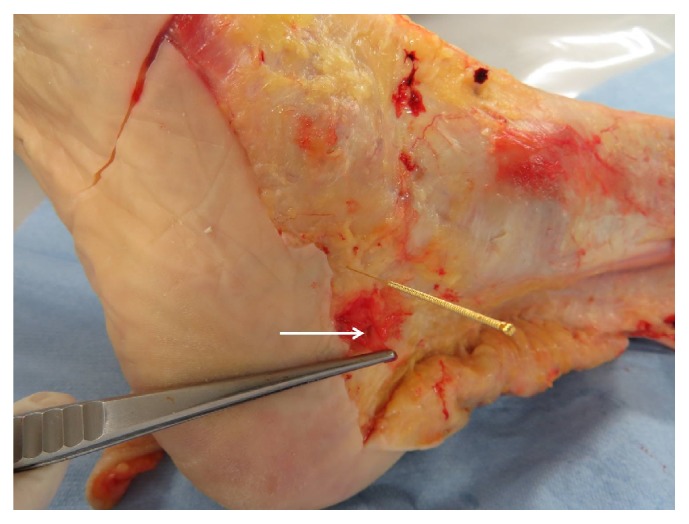
VNB without acupuncture point, acupuncture point distal to the VNB, and dorsal to the lateral malleolus sinistra.

**Figure 4:**
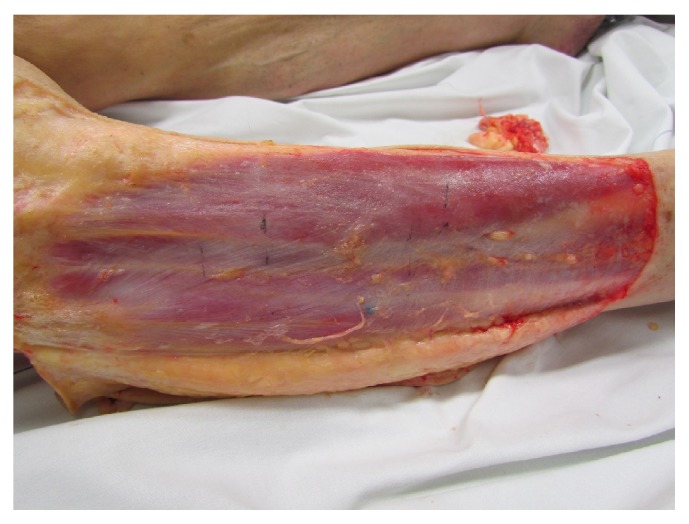
Fascia course of the stomach meridian.

**Figure 5:**
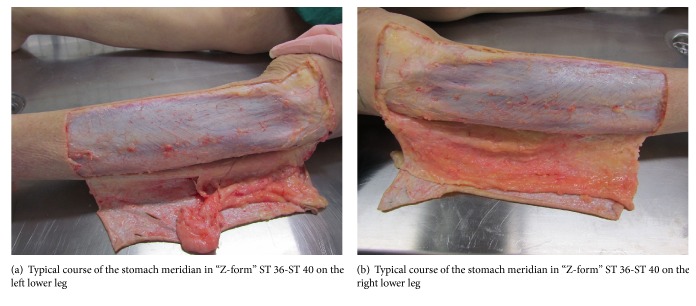


**Figure 6:**
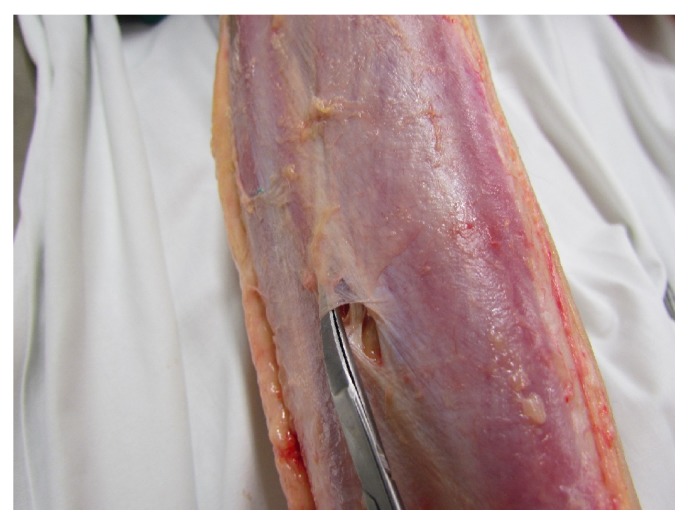
Preparatory presentation of the fascia of the stomach meridian (superficial fascia cristis sinistra).

**Figure 7:**
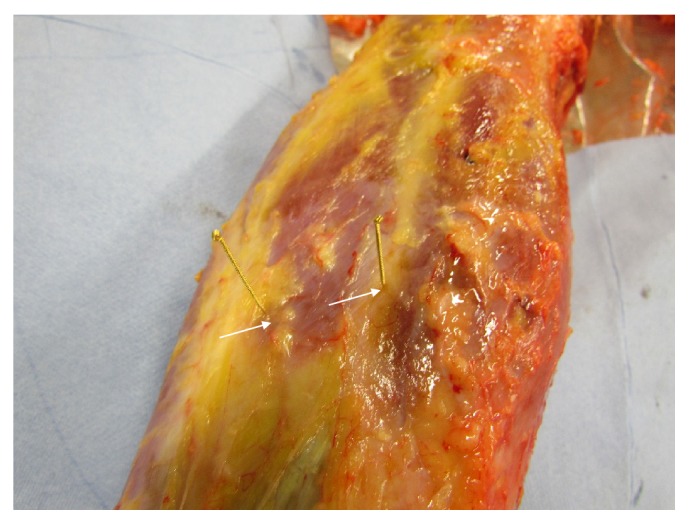
Fascia course of the gallbladder meridian between GB 35 and GB 36.

**Figure 8:**
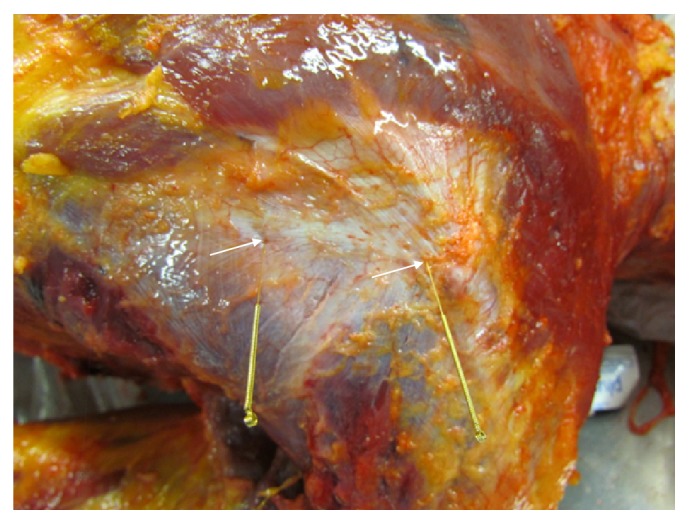
Fascia course of the small intestine meridian within the region of SI 10 to SI 11 at the fascia above the right scapula.

**Figure 9:**
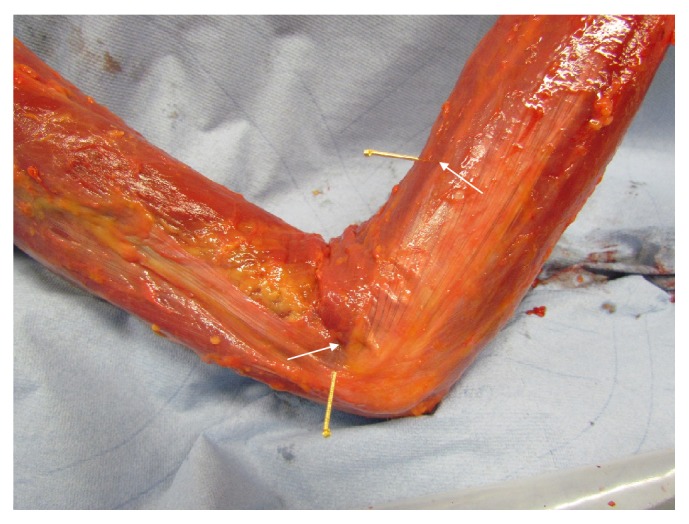
Fascia course of the large intestine meridian LI 10 to LI 11 on the right arm laterally.

**Figure 10:**
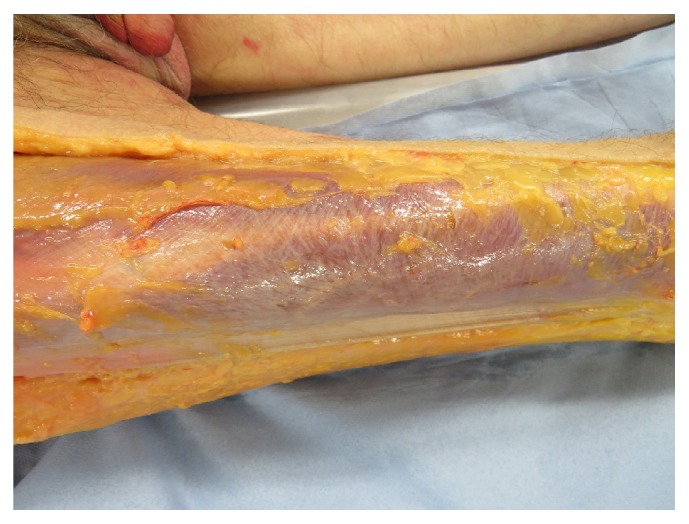
Fibre course of the fascia corporis externa on the right thigh. The fibres run at right angles to the course of the stomach meridian. The fascia lata corresponds to the course of the gall bladder meridian.

**Figure 11:**
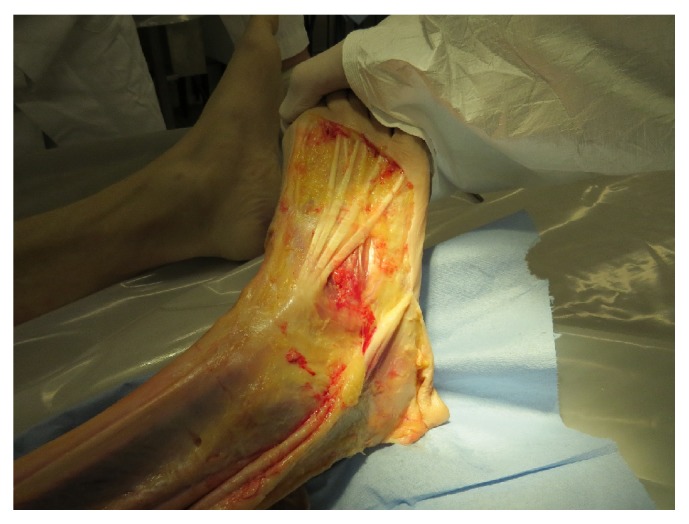
In the forefoot area, there are no structures of the fascia corporis externa that would correspond to a meridian course. Striking is the tendons of the aponeuroses tendinum extensorum digitorum pedis, which in parts follow the gastric meridian.

**Figure 12:**
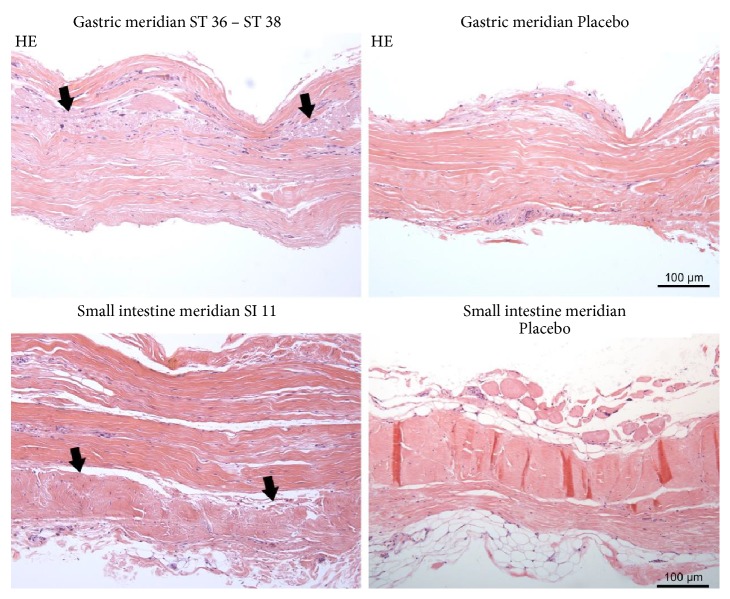
Representative microphotographs of fascia on the gastric and small intestine meridian. In the verum (left column), the change in the direction of the collagen fibres is clearly visible (arrow); this is not present in the preparation of placebo (right column).

**Figure 13:**
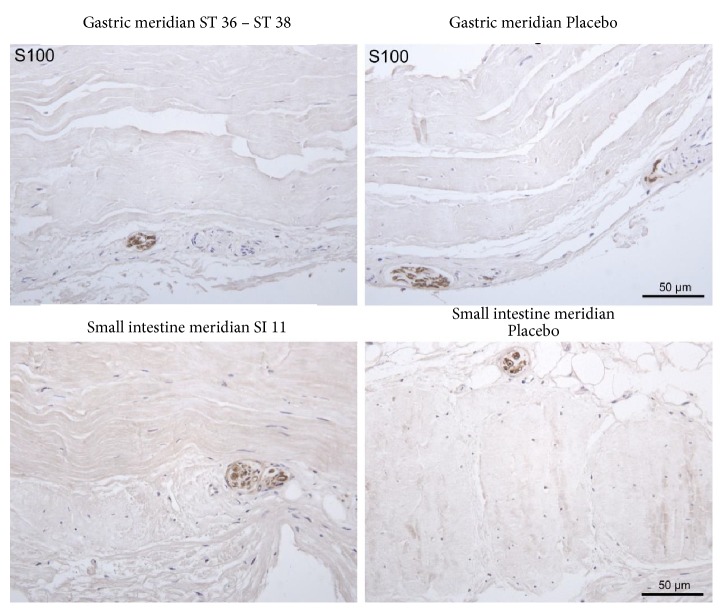
Representative photomicrograph of fascia on the gastric and small intestine meridian. Nerve fibre bundles of approximately the same calibre or vascular nerve were found in all preparations. Immunohistochemical staining with an antibody against S-100.

The main results from our anatomical and morphological investigations can be found in the photographs from Figures 1–13. In Figure 1, diverse vascular nerve bundles (VNBs) distal to the medial side of the left lower leg are depicted. In Figure 2, we can recognize a VNB at the acupuncture point and, proximal of it, we can see another VNB on the left dorsum, but without an acupuncture point nearby. In Figure 3, we see a VNB without a reference to a known acupuncture point; the next known acupuncture point lies distal to the VNB and dorsal to the lateral malleolus sinistra.

References of the detected VNBs to the anatomy of the acupuncture meridian can be established with the help of the investigations of Dr. Heine [13, 14]. Vascular nerve bundles are defined according to Heine as a structural principle of the acupuncture point. It is a perforating structure consisting of a vegetative nerve, artery, and vein, surrounded by loose connective tissue.

In Figure 4, the fascia course of the stomach meridian can be seen. In Figures 5(a) and 5(b), we can perceive the typical course of the stomach meridian in “Z-form” ST 36-ST 40 on the left (a) and right (b) lower leg.  Figure 6 represents a preparatory presentation of the fascia of the stomach meridian (superficial fascia cristis sinistra).

In Figure 7, we can discern the fascia course of the gallbladder meridian between GB 35 and GB 36

In Figure 8 we can see the fascia course of the small intestine meridian SI 10 to SI 11 at the fascia above the right scapula. In Figure 9, we can discern the fascia course of the large intestine meridian LI 10 to LI 11 on the right arm laterally.

Figure 10 shows that the structures of the external fascia corporis cross the course of the gastric meridian at right angles at the thigh. The fascia lata would correspond to the course of the gallbladder meridian.

Figure 11 shows that at the forefoot no courses of the fascia corporis externa corresponding to the gastric meridian can be found. The aponeuroses tendinum extensorum digitorum pedis follows in one of its parts the course of the gastric meridian.

In Figure 12, representative microphotographs of fascia on the gastric and small intestine meridian are summarised. In the verum, the change in the direction of the collagen fibres is clearly visible (arrow), which is not present in the preparation of placebo. In Figure 13, representative photomicrographs of fascia on the gastric and small intestine meridian are indicated. Nerve fibre bundles of approximately the same calibre or vascular nerve were found in all preparations.

Two sections of the same piece of fascia were photographed each time, to make it clear that the fascia superficialis is not of the same size or structure throughout. At the acupuncture point SI 11, the change in the fibre direction was particularly well represented. There was no histological difference between verum (acupuncture meridian) and placebo. Collagen 1 was found as a morphological substrate for the fascia of the meridians shown.

The fascia at the examined localisations varied in thickness and consisted of two to three layers of collagen fibres. On the samples of both the gastric meridian and the small intestine meridian, it was possible to show histologically the change of the fibre course in a connective tissue layer. This was not the case with the placebo preparation. In addition, there was no histological difference between verum (acupuncture meridian) and placebo. In all preparations, smaller nerve fibre bundles or the perivascular plexus could be detected by staining with S-100. There were no noticeable differences in the density or location of nerve fibres between the verum and the placebo.

We dissected four specimens and additionally two lower legs, in total 10 lower legs. On each shank, ten acupuncture points (5 of the stomach and 5 of the gall bladder meridian) were evaluated. Only in two of the 100 evaluated acupuncture points, we found VNBs, corresponding to 2% of cases.

## 4. Discussion

Thereby, we established a close connection of acupuncture points with structures of connective tissue. Heine has found that, in 80% of acupuncture points, a bundle of vascular nerves of soft connective tissue passes through fascia holes to the skin. The same anatomical structures were also found by Egerbacher in cattle and dogs, without specifying a percentage of the found VNBs per dissected acupuncture points [15].

By finding fascia fractions of the human extracellular matrix with a fibre run as the course of the acupuncture meridian, we suggest that the anatomical substrate of the acupuncture meridian is the fascia superficialis corporis of the human extracellular matrix as it was suggested in scientific works [16–18]. At the same time, we found that parts of muscles, tendons, and ligaments follow the meridian course (bladder meridian; large intestine meridian).

The histology showed that, between verum (acupuncture meridian) and placebo, there is no detectable difference. The fibre folding in the meridian progression could be detected macroscopically and microscopically. However, after fixation with formaldehyde 80%, the proteins of the tissue were denaturated. Therefore, further investigations on unfixed tissue samples will be performed in future projects.

In addition, we could not represent fascia of an entire acupuncture meridian. One of the reasons for this could be that, in our preparations, the dissection of tissue adhesion was not easy to perform. At the same time we found no corresponding fibre courses of the fascia superficialis corporis on the thigh and the forefoot, which would correspond to a meridian course. We therefore hypothesize that other parts of the fascia, but also tendon courses, anatomically depict the meridian course.

Our study clearly supports the view that the human body's fascia network may be the physical substrate represented by the meridians of TCM [1]. Specifically, this hypothesis is supported by our anatomical, morphological, and histological observations made in human corpses.

## 5. Conclusions

We suggest that not only fascia, especially the fascia corporis externa, but also deeper parts form the anatomical substrate of acupuncture meridians. In addition, parts of muscles, tendons, and ligaments follow the meridian course. Our observations build an anatomical basis for examining TCM principles and therapies, and it supports a holistic approach to diagnosis and treatment of diverse diseases.

Since we found VNBs in just a few of the acupuncture points and as we found VNBs even without an acupuncture point, we are no longer convinced that the sole concept of the function of the acupuncture system over neural reflexes is valid.

## Data Availability

The data used to support the findings of this study are available from the corresponding author upon request.
